# Descriptive analyses of knowledge, attitudes, and practices regarding rabies transmission and prevention in rural communities near wildlife reserves in Uganda: a One Health cross-sectional study

**DOI:** 10.1186/s41182-024-00615-2

**Published:** 2024-07-19

**Authors:** Collins G. K. Atuheire, James Okwee-Acai, Martha Taremwa, Odoch Terence, Sarah N. Ssali, Frank N. Mwiine, Clovice Kankya, Eystein Skjerve, Morten Tryland

**Affiliations:** 1 Department of Biosecurity, Ecosystems & Veterinary Public Health, College of Veterinary Medicine, Animal Resources & Biosecurity, Makerere University, P.O Box 7062, Kampala, Uganda; 2 Department of Veterinary Pharmacy, Clinical and Comparative Medicine, College of Veterinary Medicine, Animal Resources & Biosecurity, Makerere University, P.O Box 7062, Kampala, Uganda; 3 School of Women and Gender Studies, College of Humanities, Makerere University, P.O Box 7062, Kampala, Uganda; 4 Department of Biomolecular Resources and Bio-Lab Sciences, College of Veterinary Medicine, Animal Resources and Biosecurity, Makerere University, Kampala, Uganda; 5 Department of Production Animal Medicine, Norwegian University of Life Sciences, Ås, Norway; 6 Department of Arctic and Marine Biology, UiT-The Arctic University of Norway, Tromsø, Norway; 7 Department of Forestry and Wildlife Management, Inland Norway University of Applied Sciences, 2480 Koppang, Norway

**Keywords:** Rabies, Rural communities, Wildlife reserves, Knowledge, Attitudes, Practices, One Health, Uganda

## Abstract

**Background:**

Despite urban (domestic dog) rabies cycles being the main target for rabies elimination by 2030, sylvatic (wildlife) rabies cycles can act as rabies spillovers especially in settlements contiguous to wildlife reserves. Rural communities next to wildlife reserves are characterized by unique socio-demographic and cultural practices including bat consumption, hunting for bushmeat, and non-vaccination of hunting dogs against rabies among others. This study aimed to compare the knowledge, attitudes, and practices (KAPs) related to rabies transmission and prevention in the three districts of Uganda; (1) Nwoya, neighboring Murchison Falls National Park (MFNP) in the north, (2) Kamwenge neighboring Kibaale National Park (KNP), Queen Elizabeth National Park (QENP) and Katonga Game Reserve (KGR) in the west, and (3) Bukedea, neighboring Pian Upe Game Reserve (PUGR) in the east of Uganda.

**Methods:**

A community-based cross-sectional survey was conducted in settlements contiguous to these wildlife reserves. Using a semi-structured questionnaire, data were collected from 843 households owning dogs and livestock. Data were collected between the months of January and April 2023. Stratified univariate analyses by district were carried out using the Chi-square test for independence and Fisher’s exact test to compare KAPs in the three study districts.

**Results:**

The median age of study participants was 42 years (Q1, Q3 = 30, 52) with males comprising the majority (67%, *n* = 562). The key findings revealed that participants from the Nwoya district in the north (MFNP) had little knowledge about rabies epidemiology (8.5%, *n* = 25), only 64% (*n* = 187) of them knew its signs and symptoms such as a rabid dog presenting with aggressiveness and showed negative attitudes towards prevention measures (15.3%, *n* = 45). Participants in the Kamwenge district-west (KNP, QENP, and KGR) had little knowledge and negative attitude towards wildlife–human interaction pertaining to rabies transmission and prevention especially those with no or primary level of education (20.9%, *n* = 27) while participants from Bukedea in the east (PUGR) had remarkedly poor practices towards rabies transmission, prevention, and control (37.8%, *n* = 114).

**Conclusions:**

Rabies from sylvatic cycles remains a neglected public health threat in rural communities surrounding national parks and game reserves in Uganda. Our study findings highlight key gaps in knowledge, attitudes, and practices related to rabies transmission and prevention among such communities. Communication and action between veterinary services, wildlife authority, public health teams, social science and community leaders through available community platforms is key in addressing rabies among the sympatric at-risk communities in Uganda.

**Supplementary Information:**

The online version contains supplementary material available at 10.1186/s41182-024-00615-2.

## Background

Rabies is a societal neglected zoonotic disease ravaging poor and remote human settlements disproportionately relative to urban rich communities. The disease is caused by the rabies virus (RABV), a member of the genus *Lyssavirus* that belongs to the family *Rhabdoviridae* [1]. Rabies has been reported from most parts of the world. Only a few countries or regions remains rabies free, but the disease is most predominant in Asia and Sub-Saharan Africa [2–7]. In Western Europe, the red fox (*Vulpes vulpes*) is the major reservoir, while in USA, racoons (*Procyon lotor*), skunks (*Mephitis mephitis*), gray foxes (*Urocyon cinereoargenteus*), coyotes (*Canis latrans*) and bats (*Desmodus rotundus*) have been reported as main carriers [2, 8–12]. The main reservoir species in Asia include domestic dog/stray dogs (*Canis lupus familiaris*), red fox (*Vulpes vulpes*), racoon dog (*Nyctereutes procynoides*), and ferret badger (*Melogale moschata*) [13–16].

The main rabies virus carriers in Africa include the domestic dog, jackal (*Canis adustus* and *Canis mesomelas*), kudu (Namibia) (*Tragelaphus strepsiceros*) and mongoose (*Cynictis penicillata*) [17, 18]. In Uganda, the transmission of rabies via most of these reservoir species is poorly understood [19]. Communities neighboring wildlife reserves pose unique practices such as bat consumption in Murchison Falls (MFNP) and Queen Elizabeth National Park (QENP) that have the potential to expose them to bat-borne zoonotic diseases (BZD) such as rabies [20].

Humans usually contract rabies via contact with infected animals, usually via a bite through the skin or via mucous membranes. The virus enters the body where it multiplies near the point of entry and if not prevented at this point, it may invade the nerve cells and find its way to the spinal cord and the brain. The incubation period in humans varies and it can range from days to years, but the average length is 3–8 weeks [21] and the disease poses a complex clinical picture manifesting as either furious (encephalitic) or dumb (paralytic) forms both involving the brainstem resulting into diagnostic and therapeutic challenges [22].

Prevention strategies against rabies such as mass vaccination of dogs are advocated for towards elimination of rabies by 2030 [23, 24], however, most communities in developing countries have lingering awareness gaps towards pre and post-exposure vaccination against rabies. Rabies post-exposure prophylaxis (PEP) for non-previously vaccinated persons is recommended as follows: first cleansing the bite wound thoroughly with a virucidal agent such as povidone–iodine, then administering rabies immunoglobulin (RIG), followed by four Intramuscular (IM) doses, 1ML each, of rabies vaccine, Human Diploid Cell culture Vaccine (HDCV) or Purified Chicken Embryo Cell culture Vaccine (PCECV) i.e., at days 0, 3, 7 and 14 post exposure. A 5th dose on day 28 may be administered for immunocompromised persons. If someone had previously received rabies vaccine, pre-exposure prophylaxis (PrEP), RIG is not administered after exposure but will receive only two (2) IM doses of rabies vaccine at day 0 and day 3 which act as boosters [25].

Previous studies in Uganda have revealed existing gaps in knowledge, attitudes and practices towards rabies transmission and prevention especially among those with low levels of formal education. In the Masaka and Wakiso districts, Central Uganda, Kankya et al. and Kisaka et al. found poor post-wound care following a dog bite, irregular dog vaccination practices against rabies and poor health-seeking behavior following dog bites and such were noticed more among participants than had completed primary and below level of formal education [26, 27]. In Northwest and Western parts of Uganda, Omodo et al. revealed knowledge gaps about the risk of contracting rabies from stray dogs and the vaccination and handling of roaming dogs [1]. The point prevalence of rabies among domestic dogs due to interaction with wildlife has been documented at 20% in 2013 at the human–wildlife interface in western Uganda such as Mgahinga National Park (MNP), QENP and Bwindi Impenetrable Forest (BIF) [28]. Since then, during the last decade, there has been no awareness program that has been carried out in such sympatric settlements. Gaps in knowledge, practices, and attitude towards rabies transmission and prevention among Ugandan communities in proximity to wildlife-protected areas will continue to favor sylvatic rabies cycles making rabies control efforts in the domestic dog (urban) cycle futile. The aim of this study was to compare knowledge, attitudes and practices towards rabies transmission and prevention as stratified by levels of education in households surrounding selected wildlife-protected areas in Uganda.

## Methods

### Study design

A community-based cross-sectional study was carried out in communities next to national parks and game reserves between the months of January and April 2023 in three districts of Uganda. Knowledge, attitude, and practices pertaining to rabies transmission and prevention at the interface between animals, wildlife and humans were key aspects of the study. A structured questionnaire (S1) was administered by five (5) trained research assistants. The questionnaire was presented to households owning dogs (since they are primarily responsible for rabies vaccination especially dog vaccination) and livestock and that were living in proximity to wildlife reserves in three different districts of Uganda.

### Sample size and statistical power estimation

Using the modified Kish Leslie formula as [29, 30]:

$$n = \frac{{\left( {Z_{\beta } + Z_{\alpha } } \right)^{2} *P\left( {1 - P} \right)}}{{d^{2} }}*D,$$

*n* = the minimum sample size. $Z_{\alpha }$ = the standard normal deviation at 5% = 1.96; $Z_{\beta }$ = one left-tailed z statistic at the area of 20% (80% statistical power) = 0.84; *D* = design effect (using *D* = 2 according to the recommendations by WHO [29]); *P* = proportion of knowledge towards rabies (using proportion of respondents with knowledge towards rabies transmission in west nile Uganda by Omodo et al. as 16% [1, 31] and *d* = level of precision taken at 0.05.

By substitution, *n* = 843. Dog bites have been associated with the risk of rabies in rural eastern Uganda [32] and they have been found distributed as 36%, 29% and 35% in Bukedea, Kamwengye and Nwoya, respectively (Atuheire et al. 2024, unpublished). Subjecting these proportions to *N* = 843; we were able to sample *n* = 302, *n* = 245 and *n* = 296 in Bukedea, Kamwengye and Nwoya, respectively. Basing on education level, we used a sample size from the Kamwenge district since it had a majority that had no formal education (*n* = 47, 19.2%) compared to Bukedea and Nwoya districts, *p* < 0.001 (Table 1).

**Table 1:** Socio-demographic characteristics of the study population in three study sites in Uganda: rabies transmission and prevention

Variable	Overall	District			Test statistic (*p*-value)
	*N* = 843	Bukedea (*n* = 302)	Kamwenge (*n* = 245)	Nwoya (*n* = 296)	
Mean age (SD) in yrs	42.9 (15.7)	43.8 (16.1)	43.1 (15.3)	41.8 (15.5)	0.52 (0.60)^b^
Median age (Q1, Q3), skewness	42 (30, 52), 0.54	43 (30, 53), 0.53	42 (31, 53), 0.48	40 (29, 51.5), 0.59	4.71 (0.095)^c^
Sex *n* (%)					
Female	281 (33.3)	104 (34.4)	83 (33.9)	94 (31.8)	0.35 (0.84)
Male	562 (66.7)	198 (65.6)	162 (66.1)	202 (68.2)	0.18 (0.92)
Education, *n* (%)					
None	99 (11.7)	25 (8.3)	47 (19.2)	27 (9.1)	**16.4 (< 0.001)**
Primary	520 (61.7)	189 (62.6)	144 (58.8)	187 (63.2)	0.48 (0.79)
Secondary	198 (23.5)	77 (25.5)	47 (19.2)	74 (25.0)	2.74 (0.25)
Tertiary	20 (2.4)	8 (2.7)	6 (2.5)	6 (2.0)	0.25 (0.88)
University	6 (0.7)	3 (1.0)	1 (0.4)	2 (0.7)	0.66 (0.72)^a^
Religion, *n* (%)					
Seventh Day Adventist	16 (1.9)	1 (0.3)	14 (5.7)	1 (0.3)	**26.50 (< 0.0001)**
Anglican	336 (39.9)	158 (52.3)	116 (47.4)	62 (21.0)	**41.77 (< 0.001)**
Born again	99 (11.7)	43 (14.2)	24 (9.8)	32 (10.8)	2.61 (0.27)
Catholic	369 (43.8)	89 (29.5)	85 (34.7)	195 (65.9)	**51.77 (< 0.0001)**
Muslim	18 (2.1)	10 (3.3)	3 (1.2)	5 (1.7)	3.18 (0.20)
Other	5 (0.6)	1 (0.3)	3 (1.2)	1 (0.3)	2.32 (0.31)^a^
Marital status, *n* (%)					
Divorced	18 (2.1)	5 (1.7)	2 (0.8)	11 (3.7)	**5.79 (0.06)**
Married	710 (84.4)	271 (89.7)	203 (83.2)	236 (80.0)	1.85 (0.40)
Single	75 (8.9)	16 (5.3)	29 (11.9)	30 (10.2)	**7.29 (0.03)**
Widowed	38 (4.5)	10 (3.3)	10 (4.1)	18 (6.1)	2.68 (0.26)

Bold means the finding is statistically significant, i.e* p* < 0.05

^a^Fisher’s exact *p*-value

^b^*F*-test *p*-value

^c^Median test chi-square

Equality of correlation matrices between questions and across the three districts was performed using Cochran’s *Q*-test whose *p*-value was < 0.001, a suggestion that there was no correlation between questions i.e., there was no likelihood of questions attracting a yes/no response successively [33].

### Study area

**Fig. 1:**
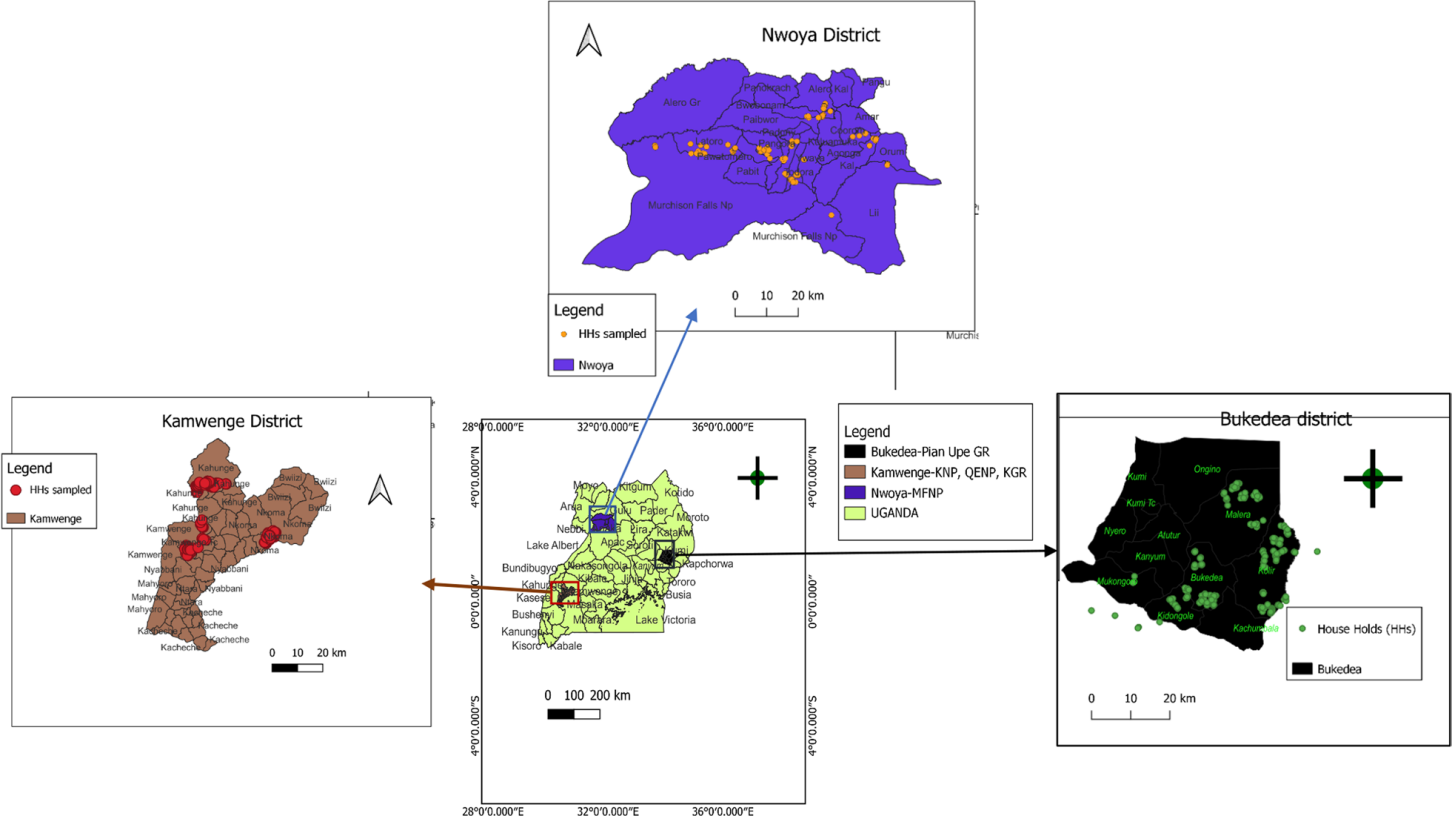
Map showing sites surrounding protected areas in Uganda. *Pian Upe GR* Pian Upe Game Reserve, *KNP* Kibaale National Park, *QENP* Queen Elizabeth National Park, *KGR* Katonga Game Reserve, *MFNP* Murchison Falls National Park

We carried out this study in three districts that neighbor wildlife-protected areas of northern (Nwoya), western (Kamwenge) and eastern (Bukedea) Uganda (Fig. 1).

In the Nwoya district (north) we studied 11 sub-counties that included Anaka, Anaka town council, Purongo, Purongo Town council, Lungulu, Koch Goma, Koch Goma Town council, Lii, Alero, Paminyai, and Got Apwoyo. The native inhabitants are Acholi whose main activity is crop and animal agriculture. Those living very close to the Murchison Falls National Park (MFNP) practice regular hunting of small animals especially antelopes and rodents using hunting dogs (i.e. local Ugandan dogs unprofessionally trained for hunting).

The Kamwenge district in western Uganda is unique in that it is surrounded by three protected wildlife areas QENP to the west, KNP to the north and KGR to the east. The inhabitants constitute over ten ethnic groups making Kamwenge multi-ethnic with varied cultural beliefs and norms. This ethnographic setting coupled with socio-economic challenges poses a big bearing on practices towards rabies prevention [34]. The main activity of the inhabitants in this region ranges from small-scale businesses to small-scale crops and livestock farming.

In Bukedea district in the east, six sub-counties were studied: Bukedea Trading centre, Kocheka, Kidongole, Aminit, Kamutur, and Kangole. The common husbandry practice in the Bukedea district is communal grazing where animals are left to freely graze in a common graze-land (“Aaro”). Cows, sheep and goats are often left in Aaro for many weeks without necessarily the herdsman’s attendance, but instead left to be taken care of by the dogs for protection against wild animals, especially the jackals. Hunting is another major practice in these sub counties. The native inhabitants are called Itesots (descendants of Karamojong) whose main traditional occupation is cattle keeping.

### Study population and selection criteria

This study was carried out among household members (from households contiguous to wildlife-protected areas) of Bukedea, Kamwenge and Nwoya districts of Uganda between the months of January 2023–April 2023. Households that keep dogs or have been keeping dogs for the previous 1 year, their dogs interact with livestock and consent to participate in the interview were included in the study. Households whose head or adult next of kin was absent at the time of data collection were excluded from the study.

### Sampling method

A multi-stage sampling was used [32]. The sub-counties from each study site were selected purposively based on proximity to the wildlife-protected area. Choice of a parish from a subcounty was also done purposively while village selection from each parish was carried out using systematic random sampling using the Local (village) council record book. From each village, a household was picked based on whether it keeps a dog or not. In household, the head of the family was chosen for answering the questionnaire. In case of absence of the family head, an adult next of kin was able to be involved. If both were absent, we could exclude that household.

### Data collection

#### Variables

**Fig. 2:**
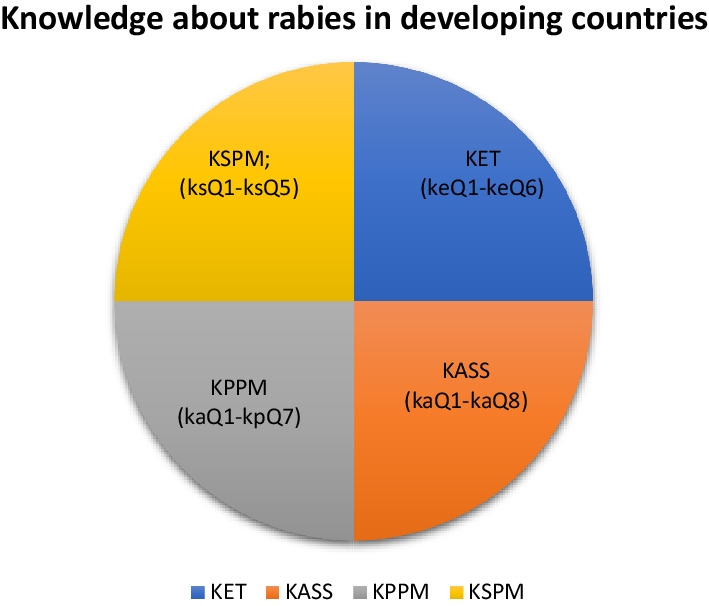
Conceptual quadrant model of knowledge about rabies

Knowledge was measured in four dimensions which we termed as rabies knowledge quadrants (Fig. 2), knowledge about proxy epidemiologic triad (KET), knowledge about signs and symptoms (KASS), knowledge about primary preventive measures (KPPM) and knowledge about secondary preventive measures (KSPM) and each comprised a set of questions adapted from U.S. CDC website concerning rabies [35].

In KET (Table 2) we considered a set of six questions ranging from keQ1 to keQ6. “keQ1: Have you heard about a disease called rabies? “keQ2: What are the main reservoirs of rabies?”; “keQ3: What are the species most affected by rabies?”; “keQ4: Is the common mode of rabies transmission a rabid dog, cat, or fox biting a human being?”; “keQ5: What is the most prone category of people at more risk for rabies”; “keQ6: What is Incubation period of rabies in animals.”

**Table 2:** Knowledge about epidemiologic triad (KET) towards rabies transmission and prevention among households neighboring national parks in Uganda

Variable	Overall	District				Education level^b^			
	*N* (%)	Bukedea (*n* = 302)	Kamwenge (*n* = 245)	Nwoya (*n* = 296)	*p*-value	Primary and below (*n* = 619)	Post primary (*n* = 224)	*N* (%)	*p*-value
Have you heard about of a disease called rabies? keQ1 *n* (%)									
No	49 (5.8)	12 (3.97)	12 (4.90)	25 (8.45)	**0.06**	12 (6.3)	0 (0.0)	12 (4.9)	**0.037**
Yes	794 (94.2)	290 (96.0)	233 (95.1)	271 (91.6)	0.84	179 (93.7)	54 (100.0)	233 (95.1)	0.24
What are the main reservoirs of rabies? keQ2 *n* (%)									
Dog and jackal and cat	27 (3.2)	23 (7.62)	4 (1.63)	0 (0.0)	**< 0.001**	4 (2.1)	0 (0.0)	4 (1.6)	0.23
Dog	476 (56.5)	74 (24.50)	106 (43.3)	296 (100.0)	**< 0.001**	76 (39.8)	30 (55.6)	106 (43.3)	0.69
Dog and cat	69 (8.2)	65 (21.52)	4 (1.6)	0 (0.0)	**< 0.001**	2 (1.1)	2 (3.7)	4 (1.6)	0.29
Dog and jackal	84 (10.0)	66 (21.85)	18 (7.4)	0 (0.0)	**< 0.001**	17 (8.9)	1 (1.9)	18 (7.4)	**0.044**
Jackal	113 (13.4)	0 (0.0)	113 (46.1)	0 (0.0)	**< 0.001**	92 (48.2)	21 (38.9)	113 (46.1)	**0.055**
I dont know	74 (8.8)	74 (24.50)	0 (0.0)	0 (0.0)	**< 0.001**	–	–	–	
The most species affected by rabies are? keQ3 *n* (%)									
I dont know	34 (4.0)	32 (10.60	0 (0.0)	2 (0.68)	**< 0.001**				
Dog	554 (65.7)	171 (56.6)	153 (62.5)	230 (77.7)	**0.005**	119 (62.3)	34 (63.0)	153 (62.5)	0.22
Dog and jackal	36 (4.3)	27 (8.94)	8 (3.27)	1 (0.3)	**< 0.001**	7 (3.7)	1 (1.9)	8 (3.3)	0.37
Dog and jackal and man	43 (5.1)	13 (4.30)	24 (9.8)	6 (2.0)	**< 0.001**	22 (11.5)	2 (3.7)	24 (9.8)	**0.043**
Dog and jackal and man and livestock	10 (1.2)	10 (3.31)	0 (0.0)	0 (0.0)	**< 0.001**				
Dog and man and livestock	28 (3.3)	5 (1.7)	6 (2.5)	17 (5.7)	**0.016**	3 (1.6)	3 (5.6)	6 (2.5)	0.19
Dog and man	122 (14.5)	41 (13.6)	47 (19.2)	34 (11.5)	**0.056**	34 (17.8)	13 (24.1)	47 (19.2)	0.87
Jackals	5 (0.6)	0 (0.0)	5 (2.0)	0 (0.0)	**0.002**	4 (2.09	1 (1.9)	5 (2.0)	0.74
Man	11 (1.3)	3 (1.0)	2 (0.8)	6 (2.0)	0.395	2 (1.1)	0 (0.0)	2 (0.8)	0.40
The common mode of rabies transmission is rabid dog, cat, or fox biting a human being keQ4 *n* (%)									
No	133 (15.85)	19 (6.33)	32 (13.17)	82 (27.7)	**< 0.001**	29 (15.3)	3 (5.6)	32 (13.2)	**0.028**
Yes	706 (84.2)	281 (93.67)	211 (86.8)	214 (72.3)	**0.019**	160 (84.7)	51 (94.4)	211 (86.8)	0.43
What is the most prone category of people to rabies? keQ5 *n* (%)									
I do not know	143 (17.0)	26 (8.61)	32 (13.1)	85 (28.7)	**< 0.001**	29 (15.2)	3 (5.6)	32 (13.1)	**0.028**
Children 1–15yrs	591 (70.1)	244 (80.8)	178 (72.7)	169 (57.1)	**< 0.001**	139 (72.77)	39 (72.2)	178 (72.7)	0.16
Men	54 (6.4)	19 (6.3)	8 (3.3)	27 (9.1)	**0.028**	5 (2.6)	3 (5.6)	8 (3.3)	0.48
Women	55 (6.5)	13 (4.3)	27 (11.0)	15 (5.1)	**0.004**	18 (9.4)	9 (16.7)	27 (11.0)	0.43
Incubation period of rabies in animals keQ6 *n* (%)									
I dont know	384 (45.6)	148 (49.0)	96 (39.2)	140 (47.3)	0.21	76 (39.8)	20 (37.0)	96 (39.2)	0.20
< 3 weeks	431 (51.1)	150 (49.7)	145 (59.2)	136 (46.0)	0.09	112 (58.6)	33 (61.1)	145 (59.2)	0.30
3–12 weeks	19 (2.3)	2 (0.7)	3 (1.2)	14 (4.7)	**0.002**	3 (1.6)	0 (0.0)	3 (1.2)	0.30
> 12 weeks–1 yr	9 (1.1)	2 (0.7)	1 (0.4)	6 (2.0)	0.13	0 (0.0)	1 (1.9)	1 (0.4)	0.10

Bold means the finding is statistically significant, i.e* p* < 0.05

^a^Fisher’s exact *p*-value

^b^Kamwenge

“keQ1: Have you heard about a disease called rabies? “keQ2: What are the main reservoirs of rabies?”; “keQ3: What are the most species affected by rabies?”; “keQ4: What the common mode of rabies transmission is rabid dog, cat, or fox biting a human being”; “keQ5: What is the most prone category of people to rabies”; “keQ6: What is Incubation period of rabies in animals”

In KASS (S2) we considered 8 questions ranging from kaQ1 to kaQ8.

“kaQ1: Does the rabid dog become aggressive and excitable?”; “kaQ2: Does the rabid dog becomes paralytic”; “kaQ3: Is There difficulty in swallowing i.e., like it has swallowed a bone”; “kaQ4: What is the most common period of communicability in dogs/cats”; “kaQ5: What is the typical incubation period in humans; “kaQ6: Are there symptoms of flue in humans”; “kaQ7: Are there confusion, agitation, anxiety in humans”; “kaQ8: Is there abnormal behaviour like delirium, hallucinations, hydrophobia, insomnia in humans”.

In KPPM (S3), a set of 7 questions were considered ranging from kpQ1 to kpQ7—“kpQ1: Does visiting a veterinary doctor regularly help control rabies?”; “kpQ2: Does vaccinating dogs annually help control rabies”; “kpQ3: Does controlling straying dogs help control rabies”; “kpQ4: Does stop allowing dogs to go to national park help control rabies”; “kpQ5: Does stop grazing in national parks help control rabies”; “kpQ6: Not getting in contact with wildlife help control rabies”; “ kpQ7: Not getting in contact with bats help control rabies”.

In KSPM (S4), we used a set of 5 questions ranging from ksQ1 to ksQ5—“ksQ1: Does washing animal bite wound with water and soap help control rabies?”; “ksQ2: Does PEP (Post Exposure Prophylaxis) with human rabies immunoglobulin (HRIG) at day 0 of exposure control rabies?”; “ksQ3: Does pre-exposure vaccination for at-risk people help control rabies?”; “ksQ4: Does gentle wash/irrigation of wound in water help control rabies”, “ksQ5: Does washing wound with diluted povidone–iodine help control rabies”.

For attitudes, we grouped the questions into two categories: Category A (Table 3): attitudes about wildlife–dog interaction and other possible causes. In this category, a set of questions ranging from aQ1 to aQ7 included—aQ1: Think that rabies is caused by wildlife livestock human interaction; aQ2: Believe that rabies is a huge burden in Uganda; aQ3: Think that rabies affects warm-blooded animals; aQ4: Believe that rabies affects wild animals and human beings; aQ5: Think that hunting dogs are more likely to transmit rabies; aQ6: Believe that bats transmit rabies; aQ7: Think that rabies can be transmitted through aerosols.

**Table 3:** Attitudes towards wildlife dog interaction and other possible attributes related to rabies transmission and prevention among households neighbouring national parks in Uganda

Variable	*N* (%)	District			*p*-value	Education level^a^		*N* (%)	*p*-value
		Bukedea (*n* = 302)	Kamwenge (*n* = 245)	Nwoya (*n* = 296)		Primary and below (*n* = 619)	Post primary (*n* = 224)		
Think that rabies is caused by wild life livestock human interaction aQ1 *n* (%)									
Agreed	546 (64.8)	264 (87.4)	160 (65.3)	122 (41.2)	**< 0.001**	124 (64.9)	36 (66.7)	160 (65.3)	0.24
Not sure	214 (25.4)	27 (8.9)	50 (20.4)	137 (46.3)	**< 0.001**	40 (20.9)	10 (18.5)	50 (20.4)	0.29
Disagree	83 (9.9)	11 (3.6)	35 (14.3)	37 (12.50)	**< 0.001**	27 (14.1)	8 (14.8)	35 (14.3)	0.62
Believe that rabies is a huge burden in Uganda aQ2 *n* (%)									
Agreed	643 (76.5)	284 (94.0)	216 (88.5)	143 (48.5)	**< 0.001**	165 (86.8)	51 (94.4)	216 (88.5)	0.33
Not sure	154 (18.3)	9 (3.0)	24 (9.8)	121 (41.0)	**< 0.001**	21 (11.1)	3 (5.6)	24 (9.8)	0.12
Disagree	44 (5.2)	9 (3.0)	4 (1.6)	31 (10.5)	**< 0.001**	4 (2.1)	0 (0.0)	4 (1.6)	0.23
Think that rabies affects warm blooded animals aQ3 *n* (%)									
Agreed	526 (61.5)	229 (76.3)	154 (63.1)	133 (45.1)	< 0.001	116 (61.1)	38 (70.4)	154 (63.1)	0.59
Not sure	294 (35.0)	63 (21.0)	83 (34.0)	148 (50.2)	**< 0.001**	67 (35.3)	16 (29.6)	83 (34.0)	0.13
Disagree	29 (3.5)	8 (2.7)	7 (2.9)	14 (4.8)	0.33	7 (3.7)	0 (0.0)	7 (2.9)	0.11
Believe that rabies affects wild animals and human beings aQ4* n* (%)									
Agreed	613 (73.1)	274 (90.7)	168 (69.1)	171 (58.2)	**< 0.001**	130 (68.8)	38 (70.4)	168 (69.1)	0.25
Not sure	191 (22.8)	20 (6.6)	61 (25.1)	110 (37.4)	**< 0.001**	47 (24.9)	14 (25.9)	61 (25.1)	0.52
Disagree	35 (4.2)	8 (2.7)	14 (5.8)	13 (4.4)	0.21	12 (6.4)	2 (3.7)	14 (5.8)	0.30
Think that hunting dogs are more likely to transmit rabies aQ4 *n* (%)									
Agreed	588 (79.1)	258 (85.7)	174 (71.3)	156 (53.1)	**< 0.001**	132 (69.5)	42 (77.8)	174 (71.3)	0.47
Not sure	202 (24.1)	30 (10.0)	56 (23.0)	116 (39.5)	**< 0.001**	44 (23.2)	12 (22.2)	56 (23.0)	0.38
Disagree	49 (5.8)	13 (4.3)	14 (5.7)	22 (7.5)	0.28	14 (7.4)	0 (0.0)	14 (5.7)	**0.024**
Believe that bats transmit rabies aQ6 *n* (%)									
Agreed	322 (38.4)	147 (49.0)	76 (31.3)	99 (33.6)	**0.001**	56 (29.5)	20 (37.7)	76 (31.3)	0.96
Not sure	411 (49.1)	122 (40.7)	120 (49.4)	169 (57.3)	**0.014**	97 (51.1)	23 (43.4)	120 (49.4)	**0.07**
Disagree	105 (12.5)	31 (10.3)	47 (19.3)	27 (9.2)	**0.002**	37 (19.5)	10 (18.9)	47 (19.3)	0.41
Think that rabies can be transmitted through aerosols aQ7 *n* (%)									
Agreed	179 (21.4)	65 (21.7)	52 (21.3)	62 (21.0)	0.99	41 (21.6)	11 (20.4)	52 (21.3)	0.38
Not sure	444 (53.0)	115 (38.5)	136 (55.7)	193 (65.4)	**< 0.001**	107 (56.3)	29 (53.7)	136 (55.7)	0.17
Disagree	215 (25.7)	119 (39.8)	56 (23.0)	40 (13.6)	**< 0.001**	42 (22.1)	14 (25.9)	56 (23.0)	0.79

Bold means the finding is statistically significant, i.e* p* < 0.05

aQ1: Think that rabies is caused by wild life livestock human interaction; aQ2: Believe that rabies is a huge burden in Uganda; aQ3: Think that rabies affects warm blooded animals; aQ4: Believe that rabies affects wild animals and human beings; aQ5: Think that hunting dogs are more likely to transmit rabies; aQ6: Believe that bats transmit rabies; aQ7: Think that rabies can be transmitted through aerosols

^a^Kamwenge

^b^Fisher’s exact *p*-value

Category B (S5): Attitudes towards prevention strategies. In this Category, Questions ranging from bQ1 to bQ5 were asked—bQ1: Think that a person bitten by a rabid dog should seek treatment from a health facility/veterinary facility; bQ2: Believe that communities are willing to vaccinate their pets/dogs; bQ3: Do you think vaccination of dogs/pets greatly contributes to rabies control in your district; bQ4: Believe that community sensitization has not been sufficiently done in our community; bQ5: Believe that health centers should work closely with veterinary office to curb down rabies.

Practices (Table 4 [pQ1, PQ2 and pQ5] and S6 [pQ3, pQ4, pQ6–pQ8]) towards rabies transmission at the wildlife–dog–livestock–human population were measured basing on 8 variables: pQ1–pQ8. They include; “pQ1: How often do you graze your animals in game reserves or national park”; “pQ2: How often do you graze your animals with dogs”; “pQ3: The grazing system used in this community/household”; “pQ4: Do your animals graze on their own”; “pQ5: How often do you vaccinate your dogs”; pQ6: How are your dogs kept”; “pQ7: How often do your dogs hunt in the game reserve or national park”; “P8: Are there any wild life attacks in your community”.

**Table 4:** Practices towards rabies transmission and prevention among households neighbouring national parks in Uganda

Variable	*N* = 843	District			*p*-value	Education level^a^		*N* (%)	*p*-value
		Bukedea (*n* = 302)	Kamwenge (*n* = 245)	Nwoya (*n* = 296)		Primary and below (*n* = 619)	Post primary (*n* = 224)		
How often do you graze your animals in game reserves or national park pQ1 *n* (%)	*N* (%)								
Never	666 (79.0)	183 (60.6)	227 (92.7)	256 (86.5)	**< 0.001**	183 (95.8)	44 (81.5)	227 (92.7)	**0.014**
Less than 3times	2 (0.2)	1 (0.3)	0 (0.0)	1 (0.3)	0.66^b^				
3 times	12 (1.4)	4 (1.3)	0 (0.0)	8 (2.7)	**0.032**^b^				
Always	163 (19.3)	114 (37.8)	18 (7.4)	31 (10.5)	**< 0.001**	8 (4.2)	10 (18.5)	18 (7.4)	**0.005**^b^
How often do you graze your animals with dogs? pQ2 *n* (%)									
Never	582 (69.0)	192 (63.6)	165 (67.4)	225 (76.0)	0.17	133 (69.6)	32 (59.3)	165 (67.4)	**0.037**
Less than 3times	40 (4.7)	9 (3.0)	21 (8.6)	10 (3.4)	**0.005**	18 (9.4)	3 (5.6)	21 (8.6)	0.20
3 times	18 (2.1)	9 (3.0)	3 (1.2)	6 (2.0)	0.37	2 (1.1)	1 (1.9)	3 (1.2)	0.79^b^
Always	203 (24.1)	92 (30.5)	56 (22.9)	55 (18.6)	**0.011**	38 (19.9)	18 (33.3)	56 (22.9)	0.35
How often do you vaccinate your dogs pQ5 *n* (%)									
3 months	54 (6.4)	26 (8.6)	6 (2.5)	22 (7.4)	**0.012**	4 (2.1)	2 (3.7)	6 (2.5)	0.71^b^
Annually	447 (53.0)	135 (44.7)	164 (66.9)	148 (50.0)	**0.001**	128 (67.0)	36 (66.7)	164 (66.9)	0.18
Never	342 (40.6)	141 (46.7)	75 (30.6)	126 (42.6)	**0.011**	59 (30.9)	16 (29.6)	75 (30.6)	0.30

Bold means the finding is statistically significant, i.e* p* < 0.05

pQ1: How often do you graze your animals in game reserves or national park; pQ2: How often do you graze your animals with dogs; pQ3: The grazing system used in this community/household

pQ4: Do your animals graze on their own; pQ5: How often do you vaccinate your dogs; pQ6: How are your dogs kept; pQ7: How often do your dogs hunt in the game reserve or national park

pQ8: Are there any wildlife attacks in your community

^a^Kamwenge

^b^Fisher’s exact *p*-value

#### Spatial and statistical analysis

We used Quantum GIS 3.24.1 free software to draw maps indicating study areas [36]. The Ugandan shape file data was extracted from diva-gis.org/gdata and imported into GIS to obtain layer maps to work with and administrative units, districts, and study sites were added to the map.

After establishing the database and cleaning data in Excel, data were transferred to Stata (SE for Windows, ver 14.2, StataCorp. College Station, TX, USA) for further statistical analyses. For sociodemographic characteristics, stratified descriptive statistics by district were carried out. For continuous variables like age, we used a one-way ANOVA (*F*-test) to compare means; we used a median test to compare age medians; Chi-square for independence of proportions test was performed for categorical variables (sex, education, religion and KAPs variables) and was carried out for each individual variable row versus district and education as columns (using stata command):

$$\hbox{``}{\varvec{chitesti}} \;{\varvec{ni}}_{1 } \;{\varvec{ni}}_{2 } {\varvec{ni}}_{3 } \backslash {\varvec{Ti}}_{. } *({\varvec{T}}._{{{\mathbf{1}} }} /{\varvec{T}}_{{{\varvec{g}} }} ) \;{\varvec{Ti}}_{. } *({\varvec{T}}._{{{\mathbf{2}} }} /{\varvec{T}}_{{{\varvec{g}} }} ) \;{\varvec{Ti}}_{. } *({\varvec{T}}._{{{\mathbf{3}} }} /{\varvec{T}}_{{{\varvec{g}} }} )\hbox{''}$$

where ${\text{ni}}$ is number of individuals in the $i{\text{th}}$ row; ${\text{Ti}}_{. }$ is the sum of individuals in the $i{\text{th}}$ row; $T._{1}$ is sum of individuals in column 1; $T._{2}$ is sum of individuals in column 2; $T._{3}$ is sum of individuals in column 3; $T_{{\text{g}}}$ is the total sample size.

Knowledge, Attitude and Practices attributes were analyzed using frequencies and percentages and chi-squared *p*-values obtained across district (study site) strata. An additional stratum by education (despite the inherent effect of one’s education level on knowledge concerning any attribute of interest; studies by Kisaka et al. and Atuheire et al. have shown a significant association between participants education level and practices towards rabies prevention in central and eastern Uganda, respectively [27, 32]) was used for Kamwenge district as the significant majority (*n* = 47, 19.2%) were found in this district compared to the rest of the districts, *p* < 0.001.

## Results

### Socio-demographic characteristics of study participants from Bukedea, Kamwenge and Nwoya districts, Uganda

Following Table 1, the median age for the participants was 42 years with lower and upper quartile at 30 and 52, respectively, and males constituted the majority (67%, *n* = 562). Most of them (62%, *n* = 520) had completed primary-level education. However, many of them from Kamwenge (19.2%, *n* = 47) had no formal education relative to other sites (*p* < 0.001).

### Knowledge about proxy epidemiologic triad (KET) towards rabies transmission and prevention among households neighboring national parks in Uganda

As shown in Table 2, participants from Nwoya district had less knowledge concerning the epidemiologic triad for rabies, about 9% (*n* = 25) of the participants in Nwoya have never heard about rabies relative to 5% (*n* = 12) and 4% (*n* = 12) in Kamwenge and Nwoya, respectively (*p* = 0.06), while only 72% (*n* = 214) knew the common mode of rabies transmission to be a rabid dog, cat, or fox biting a human in comparison to 87% in Kamwenge and 94% in Bukedea (*p* = 0.019). Only fifty-seven percent (*n* = 169) of the participants in Nwoya knew that the most at-risk category of people to rabies were children aged 1–15 years relative to 73% (*n* = 178) in Kamwenge and 81% (*n* = 244) in Bukedea (*p* < 0.001).

### Knowledge about signs and symptoms (KASS) towards rabies transmission and prevention among households neighboring national parks in Uganda

As shown in the supplementary file (S2), participants from Nwoya had little knowledge about the signs and symptoms of rabies relative to other sites (*p* < 0.001). Only, 64% (*n* = 187) of the study participants in the Nwoya district knew that a rabid dog becomes aggressive compared to 78% in Kamwenge and 86% in Bukedea (*p* = 0.008) and half of them (50%, *n* = 148) knew that a dog becomes paralytic compared to participants from Kamwenge (71%, *n* = 174) and Bukedea (71%, *n* = 213) (*p* = 0.001). The symptoms of rabies among humans such as confusion, agitation and anxiety were known to only 46% (*n* = 134) of the participants from Nwoya compared to those from Kamwenge (63%, *n* = 154) and Bukedea (67%, *n* = 199), *p* = 0.002; whereas abnormal behaviour related symptoms such as delirium, hallucinations, hydrophobia, and insomnia were only known to 39% (*n* = 115) as compared to 58% and 64% in Kamwenge and Bukedea respectively, *p* < 0.001.

### Knowledge about primary preventive measures (KPPM) towards rabies transmission and prevention among households neighboring national parks in Uganda

As indicated in supplementary file (S3), many of the participants from the Bukedea district were not willing to stop their dogs from accessing the game reserve (54%, *n* = 159) compared to those from Kamwenge (37%, *n* = 91) and Nwoya (32%, *n* = 93), *p* < 0.001. In addition, 56% (*n* = 163) of the Bukedea participants were unable to stop grazing livestock in the game reserve (56%, *n* = 163) compared to those from Kamwenge (34%, *n* = 83) and Nwoya (26%, *n* = 77), *p* < 0.001. Similar observation is noted among participants showing unwillingness to stop contact with wildlife (53%, *n* = 154) relative to those from Kamwenge (36%, *n* = 88) and Nwoya (30%, *n* = 89), *p* < 0.001.

### Knowledge about secondary preventive measures (KSPM) towards rabies transmission and prevention among households neighbouring national parks in Uganda

The findings as shown in supplementary file (S4) indicate that participants from the Bukedea district had little knowledge about secondary preventive measures (KSPM). Only 22% (*n* = 64) of the participants knew that washing animal wound with water and soap is necessary for rabies prevention compared to those from Nwoya (67%, *n* = 198) and Kamwenge (23%), *p* < 0.001. It was a similar observation for the use of povidone iodine (15%, *n* = 41) in relation to participants from Kamwenge (24%, *n* = 58) and Nwoya (53%, *n* = 162), *p* < 0.001.

### Attitudes towards wildlife dog interaction and other possible attributes concerning rabies transmission and prevention among households neighbouring national parks in Uganda

As shown in Table 3, households in Kamwenge district, disagreed that; rabies is caused by wildlife–livestock–human interaction (14%, *n* = 35) as compared to those from Bukedea (4%, *n* = 11) and Nwoya (13%, *n* = 37), *p* < 0.001. Perceptibly, 51% (*n* = 97) of the participants from Kamwenge did not believe that getting in contact with bats is a potential risk for rabies compared to their counterparts who had acquired post-primary education (43%, *n* = 23), *p* = 0.07. About seven percent (7.4%, *n* = 14) of the participants with primary and below level of education in Kamwenge, disagreed that hunting dogs have a higher likelihood of transmitting rabies relative to their counterparts who acquired post-primary education (0.0%, *n* = 0).

### Attitudes towards rabies transmission and prevention among households neighbouring national parks in Uganda

As depicted in supplementary file (S5), the Nwoya participants were not sure whether a person bitten by a rabid dog should seek treatment from a health facility or veterinary facility (15%, *n* = 45) compared to participants from Kamwenge (11%, *n* = 26) and Bukedea (3%, *n* = 8), *p* < 0.001. Eight percent (8%, *n* = 22) of the participants from Nwoya were not sure whether vaccination (in case of free vaccination campaigns) of dogs or pets contributes to the control of rabies relative to those from Bukedea (1%, *n* = 2) and Kamwenge (5%, *n* = 11), *p* < 0.001. Additionally, the Nwoya participants (5%, *n* = 14) were no sure whether health centers should work closely with the veterinary office to control rabies relative to participants from Bukedea (1.3%, *n* = 4) and Kamwenge (4%, *n* = 9), *p* = 0.06. Discernibly, 12% (*n* = 36) agreed that community sensitization towards rabies prevention has not been sufficiently done in their community as compared to those from Bukedea (2%, *n* = 7) and Kamwenge (7.4%, *n* = 18), *p* < 0.001.

### Practices towards rabies transmission and prevention among households neighbouring national parks in Uganda

As shown in Table 4, Bukedea district participants (38%, *n* = 114) were always grazing animals in the game reserve relative to those from Nwoya (11%, *n* = 31) and Kamwenge (7%, *n* = 18), *p* < 0.001. Additionally, the majority of Bukedea participants (31%, *n* = 92) often graze livestock with dogs as compared to those from Nwoya (19%, *n* = 55) and Kamwenge (23%, *n* = 56), *p* = 0.011. Close to half of Bukedea participants (47%, *n* = 141) reported to have never vaccinated their dogs as compared to participants from Nwoya (43%, *n* = 126) and Kamwenge (31%, *n* = 75), *p* = 0.011. The rest of the notable practices are depicted in supplementary file (S6).

## Discussion

Except for a study by Atuheire et al., no further rabies studies in Uganda have looked at settlements proximal to wildlife reserves where the risk of rabies and other zoonotic diseases are highly likely [32]. Our study findings indicate that rabies is poorly understood by people in the settlements contiguous to national parks and wildlife game reserves in the Nwoya, Kamwenge and Bukedea districts of Uganda.

The key findings revealed that the study participants from Nwoya district (northern Uganda, MFNP) had little knowledge about rabies epidemiological triad, signs, and symptoms for rabies, and had negative attitude towards rabies transmission and prevention.

The uniqueness of this region as compared to Kamwenge and Bukedea can be pointed to social disruption that has taken place since late 1980’s to early 2000’s by a rebel group [The Lord’s Resistance Army (LRA)] that made households so scattered, a phenomenon that hinders public health awareness campaigns to date [37]. A near study by Omodo et al. was carried out in north-west Uganda where they reported that 10% of the participants were less knowledgeable about rabies [1]. The similarities could be shared partly due to (1) they are remote and (2) were both disrupted by the LRA insurgency.

Another knowledge gap in Nwoya district was concerning reservoirs for rabies transmission where only 78% of the households knew that either a rabid cat, fox, jackal, or dog could transmit the disease. Our findings are comparable to those reported in Kigali Rwanda by Ntampaka et al. where they found that 74% knew about human-rabies transmission through rabid dog bites [38]. The similarity could be due to awareness programs by individuals that possess communication channels like radios.

Only fifty-seven percent of households in Nwoya recognized that the most at-risk category of persons for rabies are children aged below 15 years. Studies elsewhere corroborate that this age category is at relatively higher risk than the rest. A study by Kisaka et al. in urban central Uganda reports a 23% higher prevalence of rabies among children aged less than 15 years [27]. The same findings are reported in a 5 year retrospective review of records in five sub counties of Kenya i.e., Kilifi, Kisumu, Kitui, Machakos and Nandi where Ngugi et al. found 36% of rabies cases occurring among children aged below 15 years [39]. It is therefore necessary to sensitize households from Nwoya regarding the protection of children aged 15 years and below against dog bites (rabies).

Knowing symptoms and signs of rabies is fundamental to immediate response towards rabies control and prevention. Less than half of the participants in Nwoya knew the symptoms and signs of rabies in either a rabid dog or a human being. Mapatse et al. reported similar findings at the wildlife–human interface in Limpompo National Park in Mozambique where only 33% of the households knew that rabid dogs get aggressive [40]. The similarity of the findings may be explained by similar settings that share socio-ecological and economic intricacies that deter awareness programs by the government/responsible bodies [41].

In addition, 53% of the participants from Nwoya district did not know the incubation period and the common period of communicability of dog-mediated rabies. This can greatly hinder one’s seeking immediate post-exposure prophylaxis for rabies. Most studies report the incubation period for rabies to average between 10 and 180 days in dogs and 15–90 days or a year and beyond in humans, but once signs and symptoms appear, it becomes irreversibly fatal [42–45]. Accurate knowledge about the incubation period is necessary for prompt and timely seeking of rabies Post-exposure prophylaxis (PEP) [46].

Concerning attitude in Nwoya, fifteen percent of the households were reluctant about whether a person bitten by a rabid dog should seek treatment from a health facility or veterinary facility and did not support that medical personnel should work with veterinarians in rabies control and prevention. Additionally anti rabies PEP is located over 60kms away in Gulu referral hospital, a factor that limits awareness and accessibility and thus affecting attitude in this setting. Whereas studies have shown improved health service delivery and utilization such as antenatal care and childbirths in Northern Uganda due to post-war humanitarian aid, rabies has stayed neglected despite its huge fatality and public health importance [47, 48]. Similar findings have been reported in Ngorongoro and Serengeti districts of Tanzania where 25% of the participants never went for anti-rabies PEP due to distance to health facility, cost and under appreciation of animal bite risk [49].

Kamwenge (western Uganda, QENP, KNP, KGR) participants did not know about the main reservoirs for rabies such as bats and had negative attitude towards wildlife–dog interaction as possible causes of rabies transmission. Additionally, they had little knowledge of pre and post-rabies exposure prophylaxis. The lower levels of education adversely affected their knowledge and attitude aspects. Kamwenge district is a multiethnic division heavily burdened by the with- in immigrants and refugees from the Democratic Republic of Congo (DRC), a factor that makes the population dynamic and socio-economically unviable facilitating many children not schooling among other challenges [50]. Bat-transmitted rabies in Africa was first described in 1956 in Lagos Nigeria from an African straw-colored fruit bat and since then bat-borne rabies have been reported in neighboring countries such as Democratic Republic of Congo (DRC) and Kenya [51]. Lack of knowledge about the risk of rabies due to contact with bats is quite dangerous for people from Kamwenge since it borders DRC. Similar findings have been reported in Moyamba district in Southern Sierra Leone where only 28% of the participants knew about bat-borne zoonoses (BZD) [52]. Kamwenge district in Uganda and Moyamba district in Sierra Leone share hills and tropical rain forests in common that act as natural habitats for bats.

Participants in the Kamwenge district had little knowledge of pre-exposure vaccination for at-risk people (59%) as well as on post-exposure prophylaxis with Human Rabies Immune Globulin (HRIG) at day 0 of exposure (72%). This is very dangerous for this population that is situated between three wildlife-protected areas of QENP, KNP and KGR where potential rabies reservoirs are found such as jackals and stray dogs. A Study in Dakar Senegal by Mamadou et al. show similar findings that uptake of RIG was below WHO recommendation (55%) and this was attributed to the low level of education of the family head [53].

In Bukedea, eastern Uganda, the participants were not aware of the potential rabies risk associated with herdsmen, dogs and livestock getting access to the wildlife game reserve as compared to other study sites of Kamwenge and Nwoya. People from Bukedea are called Itesots and have got their heritage in livestock rearing/pastoralism as an extension of their Karamojong ancestors. Staying for long hours to days in grazelands “Aaro” is a potential attribute that denies them access to public health awareness programs [32]. Elsewhere, the interaction of dogs with wildlife (wild dogs) has been associated with increased rabies seropositivity among domestic dogs in Moshi, Tanzania at the interface of Mt. Kilimanjaro [54]. This suggests interaction with wildlife is likely to threaten humans and livestock that are found in their proximity with rabies if stringent barriers are not put in place. Similar findings have been reported by Osman et al. about rabies occurrence in cattle, sheep, goats and camel among the pastoralist community of Adadle district of the Somali region of Ethiopia [55]. Furthermore, findings reported by Tschopp et al. in the pastoralist community of Awash Basin, Eastern Ethiopia show that 79% of the pastoralists did not know how livestock acquire rabies [56]. Additionally, pastoralist communities have been found to have the greatest number of dogs used for grazing and are characterized by poorly resourced medical facilities located at far distances [57]. This avalanche of exposure factors put pastoralist people at more risk of rabies and other neglected tropical diseases than the rest of the population.

Furthermore, participants from Bukedea district had little knowledge about secondary prevention measures for rabies especially concerning post animal bite wound care with water and soap or povidone iodine. A closer study in Wakiso urban central Uganda by Kisaka et al. reported non-compliance with preclinical management of bite wounds among post-animal bite victims with only 19% applying the recommended substances [27]. The WHO recommends flushing of bite wounds with water followed by povidone–iodine or other available antiseptic [58]. Coupled with long distances to the nearby health facility for rabies care, the prognostic outcomes following a rabid animal bite are most likely to be poor in rural hard-to-reach poorly resourced settings. In Bukedea, the nearest hospital with anti-rabies services is Atutur hospital situated 22 km away. A similar study done in Mozambique by Mapatse et al. at the wildlife–human interface report different findings where the majority of the participants had good knowledge about post-bite wound care [40]. The difference is explainable by dissimilar study populations where Mapatse et al. interviewed health professionals as opposed to our study which interviewed community members. Our findings provide a true picture of knowledge about post-animal bite wound care in a community setting near wildlife reserves.

Bad practices potential of transmitting rabies were noted in the Bukedea district, namely, daily grazing of animals in the Pian Upe game reserve, having never vaccinated their dogs against rabies and having all their dogs roaming. Odoch and colleagues have reported that most dogs in eastern Uganda are free roaming with an average range of 5.7 hectares per dog [59]. This is risky for rabies transmission in the region. There are no country-specific studies assessing the risk of rabies by grazing livestock in wildlife-protected areas. However, a study done in south western Uganda settlements near three national parks of Mgahinga, Queen Elizabeth and Bwindi impenetrable forest by Millan et al. has reported a 19.6% prevalence of rabies among dogs commonly interacting with wildlife through herding and hunting [28]

Participants not vaccinating their dogs could be attributed to location i.e. hard-to-reach settlements are mostly left out during poorly resourced free mass rabies vaccination that often target urban centers. In a study carried out in rural and urban regions of southern Malawi by Carlos et al. reported a lower vaccination rate in rural and far regions than in urban areas [60]. Similar findings from a vaccination campaign study have been reported in 25 districts of south-eastern Tanzania and showed that districts with a lower budget were not able to achieve the vaccination target (OR = 0.46, 95% CI 0.32–0.44) [61]. Poor resources coupled with far distances will continue to hinder the WHO rabies vaccination target of 70%. A study in a similar setting of Laikipia county in Kenya where agro-pastoralism and ecotourism are practiced reports that dog mass vaccination against rabies never reached 70% [62].

Our current study was not able to interview key informants like veterinarians, medics and wildlife officers since the target was to obtain community-based perspectives about rabies transmission and prevention. In addition, policy and regulation desirability bias for participants tending to report good practices in line with wildlife–dog–livestock interaction such as grazing and hunting was a challenge to responses given by respondents (The Uganda Wild Authority prohibits unauthorized persons from accessing the game reserve/national park). However, we explained during consent how the study is purely and intentionally for research purposes only. Furthermore, our study did not carry out logistic regression to assess the possible predictors associated with the KAPs, however, Monje et al. had shown that little knowledge towards rabies negatively affects attitudes which in turn adversely affects practices towards rabies management among animal and health practitioners in eastern Uganda with no capture of influence of proximity to wildlife reserves [31]. In addition, our study did not carry out focus group discussions and key informant interviews to explore in detail the KAPs among the study population.

Our current study has addressed a substantial set of variables (and looking at various dimensions with detailed univariate analysis across the study districts) on rabies transmission and prevention in relation to knowledge, attitude, and practices among settlements next to wildlife reserves. This is the first study in Uganda to give attention to such zoonotic interfaces regarding rabies. Our findings will inform relevant stakeholders such as Uganda ministry of health (MOH), Uganda ministry of agriculture, animal industry and fisheries (MAAIF) to collaborate on addressing rabies in seemingly neglected sympatric communities of higher rabies and other zoonotic diseases risk in Uganda.

## Conclusion

Despite rabies being highly fatal, rural human settlements contiguous to wildlife reserves grapple with little knowledge, negative attitudes, and poor practices towards rabies prevention. This implies that wildlife–human interfaces will continue to act as leakage points for rabies from the wild to the domestic dog population hence facilitating continuous rabies spillovers.

There is a need for continued rabies surveillance along wildlife–human interfaces through multidisciplinary approaches (One health) to involve stakeholders from health, veterinary, wildlife, social sciences, and community leaders. Additionally, using available and workable communication channels such as local radio stations, women and men financial group gatherings, religious service meetings, burial ceremonies etc. for rabies awareness is recommended. Key policy issues to address from our study findings include dog vaccination especially among hunters, enforcing action against grazing in the game reserve or exploring other sources of pasture such as silage, increased education about dog meat consumption and roaming dogs in sympatric communities contiguous to wildlife reserves. Finally, detailed qualitative studies involving focus group discussions and key informant interviews are necessary to explore practices towards rabies transmission and possible prevention strategies in communities contiguous to wildlife reserves in Uganda.

### Supplementary Information

Supplementary Material 1.

Supplementary Material 2.

Supplementary Material 3.

Supplementary Material 4.

Supplementary Material 5.

Supplementary Material 6.

## Data Availability

The dataset used during the current study is available from the corresponding author on reasonable request.

## Abbreviations

CAOs: Chief Administrative Officers
CIDIMOH: Climate change and Infectious diseases management, One Health approach
DVOs: District Veterinary Officers
GARC: Global Agenda for Rabies Control
HRIG: Human Rabies Immune Globulin
KASS: Knowledge about signs and symptoms
KET: Knowledge about proxy epidemiologic triad
KGR: Katonga Game Reserve
KNP: Kibale National Park
KPPM: Knowledge about primary preventive measures
KSPM: Knowledge about secondary preventive measures
MaKRIF: Makerere University Research and Innovation Fund
MFNP: Murchison Falls National Park
PEP: Post Exposure Prophylaxis
PUGR: Pian Upe Game Reserve
QENP: Queen Elizabeth National Park
SPHREC: School of Public Health Research Ethics Committee
