# Supplementary material for: Descriptive analyses of knowledge, attitudes, and practices regarding rabies transmission and prevention in rural communities near wildlife reserves in Uganda: a One Health cross-sectional study
Source: Trop Med Health. 2024 Jul 19;52:48. doi: 10.1186/s41182-024-00615-2 (PMC11264860; doi:10.1186/s41182-024-00615-2)

Rabies: Prevalence & KAPs Project at Wild-life Animal Human Interface (KAPs-WAH)

Questionnaire Identification

A1Questionnaire ID:

____

A1.1Name of enumerator
Put your initials say for AM: meaning Arthur Mirimu

____

A2District name:

- [ ] Bukedea
- [ ] Nwoya
- [ ] Kawengye
- [ ] other
- [ ] Option 5

A3Sub-county name:

- ○ Kangole
- ○ Kamutur
- ○ Aminit
- ○ Bukedea sub-county
- ○ Bukedea TC
- ○ Malera
- ○ Other

A3.1if other sub-county, specify

____

A4Parish name:

____

A5Village Name:

____

A6location of the village-Ecological

- ○ near a game reserve/national park
- ○ away from a game reserve/national park
- ○ with in the game reserve

A7Location of village

- Rural
- Urban
- Semi-rural
- Semi-urban

A8Name of House Hold head (HHH)

____

A9Cell phone contact

____

A10Other than HHH, who are you

- Son
- Daughter
- Wife
- Brother
- Sister
- Other

Socio-demographic characteristics of study participants

s1age in completed years

____

s2Sex

- Male
- Female

s3Level of education

- none
- primary
- secondary
- tertiary but university
- university

s4religion

- anglican
- catholic
- muslim
- SDA
- born again
- other

s5other religion

____

s6tribe

- Itesot
- Acholi
- Lango
- mutooro
- K`ajong
- mukiga
- munyankole
- muhima
- other

s7other tribe

____

s7Marital status

- Married
- Divorced
- Widowed
- Single
- Other

s8Other marital status

____

s9Smoke

- no
- yes

s10How many cigars per day

____

s11Alcohol consumption

- no
- yes

s12Which type of alcohol

- Waragi
- Beer
- Toonto
- ajono
- other

s14Amount of alcohol you consume daily in bottles

____

» Group

» » Risk factors for Rabies at Wild life -Animal- Human interface

R1Who fetches fire wood

- Husband
- Wife
- Children
- Other

R2Do you obtain firewood from Game reserve/National park

- Yes
- No

R3Distance from firewood source in kms

____

R4Who fetches water?

- husband/father
- mother
- children
- other

R5Do you obtain water from Game reserve /national park?

- Yes
- No

R6Distance from water source kms?

____

R7Are there stray dogs that frequent this household/homestead

- ◯ yes
- ◯ no

R8Are the stray dogs vaccinated ?

- ◯ yes
- ◯ no
- ◯ I don't know

R9Do you own dogs in this household

- ◯ yes
- ◯ no

R10If yes, how many dogs

____

R10.1do you often get your dogs

- ☐ inherited from parents
- ☐ buy from neighbour
- ☐ buy from another sub-country
- ☐ buy from another district
- ☐ I pick them from the bush/wild
- ☐ I pick homeless dogs
- ☐ buy from another country
- ☐ any other source

R11if other source, specify

____

R12Are your dogs vaccinated against rabies

- ◯ yes
- ◯ no
- ◯ i don't know

R13how long time ago (months) did they receive the last shot of rabies

____

R14Are your dogs used for hunting

- ◯ Yes
- ◯ No

R15How many times do you hunt per month

____

R16Do you hunt from game reserve/national park

- ◯ yes
- ◯ no
- ◯ other hunting place

R17if other hunting place, which one

____

R18Which other animals do you have

- ☐ cattle
- ☐ sheep
- ☐ goats
- ☐ pigs
- ☐ monkeys
- ☐ other

R19if you have other animals, what are they

____

R20Are there straying wild animals in this community

- ◯ yes
- ◯ no

R21If yes for straying animals, what are they

- ☐ leopards
- ☐ lion
- ☐ cheetah
- ☐ foxes
- ☐ elephants
- ☐ other

R20Do you graze animals in the game reserve/national park?

- ◯ yes
- ◯ no

R21if other, which one

R22Do you own wild animals

- yes
- no

R23Which wild animals do you own

- foxes
- monkeys
- other

R24if own other wild animals, which ones

R25Are these wild animals vaccinated

- No
- Yes
- i dont know

R26Which wild animals normally interact with your dogs

- foxes
- bats
- wild cats
- other

R27if other wild animals, specify

Prevalence factors: Biological determinants

P1Have you had a confirmed dog rabies case in this household

- yes
- no

P2How was the case confirmed

- laboratory
- head of community
- veterinary officer
- medical doctor
- herbalist
- other

P3How long ago did you get the dog case in months

____

P4Have you had a confirmed human case in this household

- yes
- no

P5How was the human case confirmed

- laboratory
- head of community
- veterinary officer
- medical doctor
- herbalist
- other

P6Was there any human death due to rabies

- yes
- no

P7How long ago did you confirm human deaths in months

____

P8Have you had a confirmed livestock case in this household

- yes
- no

P9How was the case confirmed

- laboratory
- head of community
- veterinary officer
- medical doctor
- herbalist
- other

P10How long ago did you confirm live stock case in months

____

P11Which domestic animals did you confirm in this household/village

- [ ] cattle
- [ ] sheep
- [ ] goat
- [ ] other

p11.1If other domestic animals, specify

____

P12Have you confirmed a wild animal rabies case in this community

- ( ) yes
- ( ) no

P13How was the case confirmed

- [ ] laboratory
- [ ] head of community
- [ ] veterinary officer
- [ ] medical doctor
- [ ] herbalist
- [ ] other

P14Which wild animals did you confirm in this household/village

- [ ] foxes
- [ ] monkeys
- [ ] bats
- [ ] wild dogs
- [ ] other

P15other , specify

____

P16Have you had any confirmed human death due to rabies in this community

- ( ) yes
- ( ) no

P17Have you had any confirmed dog death due to rabies in this community

- ( ) yes
- ( ) no

P18Have you found foxes or wild dogs or skunks or raccoons dead in this community

- ( ) yes
- ( ) no

P19if yes to dead foxes/wild dogs, how long time ago in months

____

Knowledge about Rabies Prevention and Control

k1 Have you heard about of a disease called rabies ?

- ◯ yes
- ◯ no
- ◯ never

k2What are the main reservoirs of rabies?

- ☐ dogs
- ☐ foxes
- ☐ cats
- ☐ I don't know

k3 What is the commonest reservoir of rabies in your area?

- ◯ dogs
- ◯ foxes
- ◯ cats
- ◯ don't know

k4 The most species affected by rabies are?

- ☐ dogs
- ☐ man
- ☐ foxes
- ☐ I don't know

k5 The common mode of rabies transmission is rabid dog, cat, or fox biting a human being

- ◯ no
- ◯ yes
- ◯ I dont know

k6 What is the most prone category of people to rabies

- ☐ children 1-15yrs
- ☐ women
- ☐ men
- ☐ I don't know

k7 Incubation period of rabies in animals is

- less than 3weeks
- 3weeks-12weeks
- beyond 12weeks but upto a year
- I dont know

» Signs and symptoms in animals

» » Furious form

ss1The rabid dog becomes aggressive and excitable

- no
- yes
- I dont know

» » Dumb form

ss2The rabid dog becomes paralytic

- no
- yes
- I dont know

ss3 There is difficult in swallowing i.e like it has swallowed a bone

- no
- yes
- I dont know

ss4what is the most common period of communicability in dogs/cats

- 3-5 days
- 14 days
- 21-90 days
- I don't know

ss5 What is the typical incubation period in humans

- 2-10 days
- 2-3 months
- 1 week - 1 year
- I don't know

» Signs and symptoms in humans

» » Early signs

ssh1 Symptoms of flue

- no
- yes
- I dont know

» » Mid-term signs (cerebral disfuntion)

ssh2 Confusion, agitation, anxiety

- no
- yes
- I dont know

» » Adavanced stage signs

ssh3 Anormal behaviour like: Delirium, hallucinations, hydrophobia, insomnia

- no
- yes
- I dont know

» Primary preventive measures

» » For dogs(pd)

pd1 visiting veterinary doctor regulary

- no
- yes
- I dont know

pd2 Vaccinate dogs annually

- no
- yes
- I dont know

pd3 control straying dogs

- no
- yes
- I dont know

pd4 Stop allowing dogs to go to national park

- no
- yes
- I dont know

pd5 stop grazing in national parks

- no
- yes
- I dont know

» » For people (pp)

pp1Don`t get in contact with wild life

- no
- yes
- I dont know

pp2Dont get in contact with bats

- no
- yes
- I dont know

» Secondary prevention (post exposure)

» » For dogs: (sp)

sp1wash animal wound with water and soap

- no
- yes
- I dont know

» » For people:

sp2PEP with Human Rabies Immune Globulin(HRIG) at 0 day of exposure

- no
- yes
- I dont know

sp3Pre-exposure vaccination for at risk people

- no
- yes
- I dont know

sp4Gentle wash/irrigation of wound in water

- no
- yes
- I dont know

sp5Wash wound with diluted povidone-iodine

- no
- yes
- I dont know

C Attitude about Rabies Prevention and Control

c1Think that rabies is caused by wild life livestock human interaction

- agree
- not sure
- disagree

c2believe that rabies is a huge burden in uganda

- agree
- not sure
- disagree

c3think that rabies affects warm blooded animals

- agree
- not sure
- disagree

c4Believe that rabies affects wild animals and human beings

- agree
- not sure
- disagree

c5think that hunting dogs are more likely to transmit rabies

- agree
- not sure
- disagree

c6Believe that bats transmit rabies

- agree
- not sure
- disagree

c7think that rabies can be transmitted through aerosols

- agree
- not sure
- disagree

c8Think that a person bitten by a rabid dog should seek treatment from a health facility/veterinary facility

- agree
- not sure
- disagree

c9Believe that communities are willing to vaccinate their pets/dogs

- agree
- not sure
- disagree

c10do you think vaccination of dogs/pets greatly contributes to rabies control in your district

- agree
- not sure
- disagree

c11Believe that community sensitization has not been sufficiently done in our community

- agree
- not sure
- disagree

c12Believe that health centres should work closely with veterinary office to curb down rabies

- agree
- not sure
- disagree

P Practices on the risk of Rabies transmission

P1 How often do you graze your animals in game reserves or national park

- always
- 3 times a week
- less than 3 times a week
- never

P2 How often do you graze your animals with dogs

- ◯ Always
- ◯ 3 times a week
- ◯ less than 3 times aweek
- ◯ Never

» Grazing system used

p3The grazing system used in this community/household

- ☐ free range
- ☐ tethering
- ☐ paddock
- ☐ strip
- ☐ other

p4if other grazing system, specify

____

p5 Do your animals graze on their own

- ◯ Always
- ◯ 3 times a week
- ◯ less than 3 times a week
- ◯ never

p6How often do you vaccinate your dogs

- ◯ 3 months
- ◯ Annually
- ◯ Never

P7How often do your dogs hunt in the game reserve or national park

- ◯ Always
- ◯ 3 times a week
- ◯ less than 3times a week
- ◯ never

P8How often do you see poachers in this game reserve

- ◯ Always
- ◯ 3 times aweek
- ◯ less than 3times a week
- ◯ never

P9How often do you vaccinate children against rabies in this community

- ◯ Annualy
- ◯ 3 times a year
- ◯ never

P10How often do you fetch water in the game reserve or national park

- ◯ Always
- ◯ 3 times aweek
- ◯ less than 3times a week
- ◯ never

P11How often do you fetch firewood in the game reserve or national park

- ◯ Always
- ◯ 3times a week
- ◯ less than 3 times aweek
- ◯ never

GPS

latitude (x.y °) ______________

longitude (x.y °) ______________

altitude (m) ______________

accuracy (m) ______________

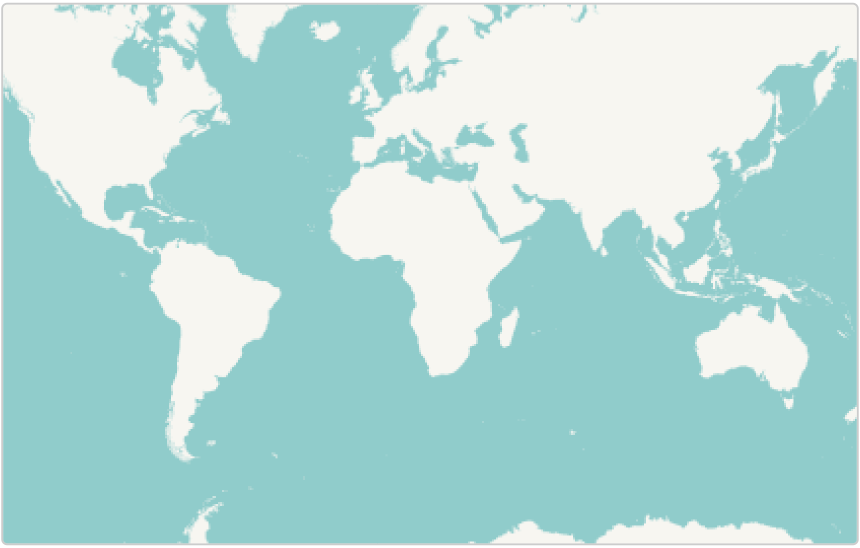

Supplement: Supplementary file 1 — Supplementary Material 1. [file 41182_2024_615_MOESM1_ESM.pdf]
