# Supplementary material for: Descriptive analyses of knowledge, attitudes, and practices regarding rabies transmission and prevention in rural communities near wildlife reserves in Uganda: a One Health cross-sectional study
Source: Trop Med Health. 2024 Jul 19;52:48. doi: 10.1186/s41182-024-00615-2 (PMC11264860; doi:10.1186/s41182-024-00615-2)
Supplement: Supplementary file 2 — Supplementary Material 2. [file 41182_2024_615_MOESM2_ESM.docx]

**Supplementary file (S2). Knowledge about signs and symptoms (KASS) towards rabies transmission and prevention among households neighboring national parks in Uganda.**

|  |  | District | | | | Education level^k^ | | | |
| --- | --- | --- | --- | --- | --- | --- | --- | --- | --- |
| Variable | N (%) | Bukedea^a^  (n=302) | Kamwenge^b^ (n=245) | Nwoya^c^  (n=296) | P-value | Primary & below (n= 619) | Post primary (n= 224) | N (%) | P-value |
| ^The rabid dog becomes aggressive and excitable.^  **kaQ1 n (%)** |  |  |  |  |  |  |  |  |  |
| No | 202(24.2) | 42(14.1) | 53(21.7) | 107(36.4) | **<0.001** | 47(24.7) | 6(11.1) | 53(21.7) | **0.012** |
| Yes | 634(75.8) | 256(85.9) | 191(78.3) | 187(63.6) | **0.008** | 143(75.3) | 48(88.9) | 191(78.3) | 0.65 |
| ^The rabid dog becomes paralytic^ **kaQ2 n (%)** |  |  |  |  |  |  |  |  |  |
| No | 301(36.0) | 86(28.8) | 70(28.7) | 145(49.5) | **<0.001** | 63(33.2) | 7(13.0) | 70(28.7) | **0.002** |
| Yes | 535(64.0) | 213(71.2) | 174(71.3) | 148(50.5) | **0.001** | 127(66.8) | 47(87.0) | 174(71.3) | 0.90 |
| ^There is difficult in swallowing i.e., like it has swallowed a bone^ **kaQ3 n (%)** |  |  |  |  |  |  |  |  |  |
| No | 415(49.6) | 169(56.7) | 102(41.8) | 144(49.0) | 0.059 | 87(45.8) | 15(27.8) | 102(41.8) | **0.007** |
| Yes | 421(50.4) | 129 (43.3) | 142(58.2) | 150(51.0) | **0.042** | 103(54.2) | 39(72.2) | 142(58.2) | 0.81 |
| ^what is the most common period of communicability in dogs/cats^  **kaQ4 n (%)** |  |  |  |  |  |  |  |  |  |
| 14 days | 40(4.8) | 14(4.7) | 8(3.3) | 18(6.1) | 0.32 | 6(3.2) | 2(3.7) | 8(3.3) | 0.92 |
| 21-90 days | 13(1.6) | 1(0.3) | 3(1.2) | 9(3.1) | **0.025** | 3(1.6) | 0(0.0) | 3(1.2) | 0.30 |
| 3-5 days | 412(49.2) | 154(51.3) | 136(55.7) | 122(41.5) | **0.049** | 105(55.3) | 31(57.4) | 136(55.7) | 0.32 |
| I don't know | 373(44.5) | 131(43.7) | 97(39.8) | 145(49.3) | 0.25 | 76(40.0) | 21(38.9) | 97(39.8) | 0.27 |
| ^What is the typical incubation period in humans^  **kaQ5 n (%)** |  |  |  |  |  |  |  |  |  |
| 1 week - 1 year | 13(1.6) | 10(3.38) | 0(0.0) | 3(1.0) | **0.005** | - | - | - |  |
| 2-10 days | 399(47.9) | 148(50.0) | 140(57.4) | 111(37.9) | **0.004** | 105(55.3) | 35(64.8) | 140(57.4) | 0.67 |
| 2-3 months | 37(4.4) | 3 (1.0) | 9(3.7) | 25(8.5) | **<0.001** | 8(4.2) | 1(1.9) | 9(3.7) | 0.29 |
| I don't know | 384(46.1) | 135(45.6) | 95(38.9) | 154(52.6) | 0.073 | 77(40.5) | 18(33.3) | 95(38.9) | 0.09 |
| ^Symptoms of flue in humans^  **kaQ6 n (%)** |  |  |  |  |  |  |  |  |  |
| No | 480(57.4) | 184(61.7) | 119(48.8) | 177(60.2) | 0.12 | 93(49.0) | 26(48.2) | 119(48.8) | 0.24 |
| Yes | 356(42.6) | 114(38.3) | 125(51.2) | 117(39.8) | **0.04** | 97(51.1) | 28(51.9) | 125(51.2) | 0.29 |
| ^Confusion, agitation, anxiety in humans^  **kaQ7 n (%)** |  |  |  |  |  |  |  |  |  |
| **No** | 349(41.8) | 99(33.2) | 90(36.9) | 160(54.4) | **<0.001** | 74(39.0) | 16(29.6) | 90(36.9) | **0.059** |
| **Yes** | 487(58.3) | 199(66.8) | 154(63.1) | 134(45.6) | **0.002** | 116(61.1) | 38(70.4) | 154(63.1) | 0.59 |
| ^Abnormal behavior like: Delirium, hallucinations, hydrophobia, insomnia in humans^  **kaQ8** n (%) |  |  |  |  |  |  |  |  |  |
| **No** | 387(46.4) | 107(36.0) | 102(41.8) | 178(60.8) | **<0.001** | 86(45.3) | 16(29.6) | 102(41.8) | **0.013** |
| **Yes** | 447(53.6) | 190(64.0) | 142(58.2) | 115(39.3) | **<0.001** | 104(54.7) | 38(70.4) | 142(58.2) | 0.96 |

^a^Pian Upe Game Reserve; ^b^Queen Elizabeth NP, Kibaale NP & Katonga game reserve; ^c^Murchison Falls NP; ^Ϯ^Fisher’s exact p-value

**^kaQ1^**^: The rabid dog becomes aggressive and excitable;^ **^kaQ2^**^: The rabid dog becomes paralytic;^ **^kaQ3^**^: There is difficult in swallowing i.e., like it has swallowed a bone;^ **^kaQ4^**^: what is the most common period of communicability in dogs/cats;^ **^kaQ5^**^: What is the typical incubation period in humans;^ **^kaQ6^**^: Symptoms of flue in humans;^ **^kaQ7^**^: Confusion, agitation, anxiety in humans;^ **^kaQ8^**^: Abnormal behavior like: Delirium, hallucinations, hydrophobia, insomnia in humans^
