# Supplementary material for: Descriptive analyses of knowledge, attitudes, and practices regarding rabies transmission and prevention in rural communities near wildlife reserves in Uganda: a One Health cross-sectional study
Source: Trop Med Health. 2024 Jul 19;52:48. doi: 10.1186/s41182-024-00615-2 (PMC11264860; doi:10.1186/s41182-024-00615-2)
Supplement: Supplementary file 3 — Supplementary Material 3. [file 41182_2024_615_MOESM3_ESM.docx]

# **Supplementary file (S3). Knowledge about Primary Preventive Measures (KPPM) towards rabies transmission and prevention among households neighboring national parks in Uganda.**

|  |  | District | | |  | Education level^k^ | |  |  |
| --- | --- | --- | --- | --- | --- | --- | --- | --- | --- |
| Variable | N (%) | Bukedea^a^ (n=302) | Kamwenge^b^ (n=245) | Nwoya^c^ (n=296) | p-value | Primary & below (n= 619) | Post primary (n= 224) | N (%) | p-value |
| ^visiting veterinary doctor regularly^  kpQ1 **n (%)** |  |  |  |  |  |  |  |  |  |
| No | 230(27.5) | 49(16.4) | 51(21.0) | 130(22.2) | **<0.001** | 40(21.2) | 11(20.4) | 51(21.0) | 0.42 |
| Yes | 605(72.5) | 249(83.6) | 192(79.0) | 164(55.8) | **<0.001** | 149(78.8) | 43(79.6) | 192(79.0) | 0.19 |
| ^Vaccinate dogs annually^ kpQ2 **n (%)** |  |  |  |  |  |  |  |  |  |
| No | 176(21.1) | 46(15.6) | 41(16.8) | 89(30.3) | **<0.001** | 29(15.3) | 12(22.2) | 41(16.8) | 0.70 |
| Yes | 657(78.9) | 249(84.4) | 203(83.2) | 205(69.7) | 0.11 | 161(84.7) | 42(77.8) | 203(83.2) | **0.058** |
| ^control straying dogs^  kpQ3 **n (%)** |  |  |  |  |  |  |  |  |  |
| No | 311(37.3) | 109(37.1) | 86(35.3) | 116(39.3) | 0.71 | 68(35.8) | 18(33.3) | 86(35.3) | 0.24 |
| Yes | 522(62.7) | 185(62.9) | 158(64.8) | 179(60.7) | 0.83 | 122(64.2) | 36(66.7) | 158(64.8) | 0.28 |
| ^Stop allowing dogs to go to national park^  kpQ4 **n (%)** |  |  |  |  |  |  |  |  |  |
| No | 343(41.2) | 159(54.3) | 91(37.3) | 93(31.5) | **<0.001** | 73(38.4) | 18(33.3) | 91(37.3) | 0.50 |
| Yes | 489(58.8) | 134(45.7) | 153(62.7) | 202(68.5) | **<0.001** | 117(61.6) | 36(66.7) | 153(62.7) |  |
| ^stop grazing in national parks^  kpQ5 n (%) |  |  |  |  |  |  |  |  |  |
| No | 323(38.9) | 163 (55.8) | 83(34.0) | 77(26.1) | **<0.001** | 66(34.7) | 17(31.5) | 83(34.0) | 0.21 |
| Yes | 508(61.1) | 129(44.2) | 161(66.0) | 218(73.9) | **<0.001** | 124(65.3) | 37(68.5) | 161(66.0) | 0.30 |
| ^Don`t get in contact with wild life^  kpQ6 n (%) |  |  |  |  |  |  |  |  |  |
| No | 331(39.8) | 154 (52.6) | 88(36.1) | 89(30.2) | **<0.001** | 70(36.8) | 18(33.3) | 88(36.1) | 0.19 |
| Yes | 501(60.2) | 139(47.4) | 156(63.9) | 206(69.8) | **0.001** | 120(63.2) | 36(66.7) | 156(63.9) | 0.32 |
| ^Don’t get in contact with bats^  kpQ7 n (%) |  |  |  |  |  |  |  |  |  |
| No | 499(60.1) | 192(65.5) | 186(76.2) | 121(41.2) | **<0.001** | 153(80.5) | 33(61.1) | 186(76.2) | **0.006** |
| yes | 332(40.0) | 101(34.5) | 58(23.8) | 173(58.8) | **<0.001** | 37(19.5) | 21(38.9) | 58(23.8) | 0.097 |

^a^Pian Upe Game Reserve; ^b^Queen Elizabeth NP, Kibaale NP & Katonga game reserve; ^c^Murchison Falls NP^,^ ^k^Kamwenge

^“kpQ1: Does visiting veterinary doctor regularly help control rabies? kpQ2: Does Vaccinating dogs annually help control rabies”; “kpQ3: Does controlling straying dogs help control rabies”; “kpQ4: Does Stop allowing dogs to go to national park help control rabies”; “kpQ5: Does stop grazing in national parks help control rabies”; “kpQ6: Not getting in contact with wild life help control rabies”; “kpQ7: Not getting in contact with bats help control rabies”^
