# Supplementary material for: Descriptive analyses of knowledge, attitudes, and practices regarding rabies transmission and prevention in rural communities near wildlife reserves in Uganda: a One Health cross-sectional study
Source: Trop Med Health. 2024 Jul 19;52:48. doi: 10.1186/s41182-024-00615-2 (PMC11264860; doi:10.1186/s41182-024-00615-2)
Supplement: Supplementary file 4 — Supplementary Material 4. [file 41182_2024_615_MOESM4_ESM.docx]

# Supplementary file (S4). Knowledge about secondary preventive measures (KSPM) towards rabies transmission and prevention among households neighbouring national parks in Uganda.

|  |  | District | | |  | Education level^k^ | |  |  |
| --- | --- | --- | --- | --- | --- | --- | --- | --- | --- |
| Variable | N (%) | Bukedea^a^ (n=302) | Kamwenge^b^ (n=245) | Nwoya^c^ (n=296) | p-value | Primary & below (n= 619) | Post primary (n= 224) | N (%) | p-value |
| ^wash animal wound with water and soap^  ksQ1 **n (%)** |  |  |  |  |  |  |  |  |  |
| No | 508(61.5) | 224(77.8) | 188(77.1) | 96(32.7) | **<0.001** | 153(80.5) | 35(64.8) | 188(77.1) | **0.014** |
| Yes | 318(38.5) | 64(22.2) | 56(23.0) | 198(67.4) | **<0.001** | 37(19.5) | 19(35.2) | 56(23.0) | 0.21 |
| ^PEP with Human Rabies Immune Globulin (HRIG) at 0 day of exposure^  ksQ2 **n (%)** |  |  |  |  |  |  |  |  |  |
| No | 526(63.9) | 176(61.8) | 176(72.4) | 174(59.0) | 0.085 | 142(75.1) | 34(63.0) | 176(72.4) | 0.078 |
| Yes | 297(36.1) | 109 (38.3) | 67(27.6) | 121(41.0) | **0.029** | 47(24.9) | 20(37.0) | 67(27.6) | 0.54 |
| ^Pre-exposure vaccination for at risk people^  ksQ3 **n (%)** |  |  |  |  |  |  |  |  |  |
| No | 421(51.2) | 127(44.7) | 145(59.4) | 149(50.7) | **0.003** | 113(59.5) | 32(59.3) | 145(59.4) | 0.22 |
| Yes | 401(48.8) | 157(55.3) | 99(40.6) | 145(49.3) |  | 77(40.5) | 22(40.7) | 99(40.6) | 0.33 |
| ^Gentle wash/irrigation of wound in water^  ksQ4 **n (%)** |  |  |  |  |  |  |  |  |  |
| No | 496(60.3) | 218(76.8) | 180(73.8) | 98(33.3) | **<0.001** | 146(76.8) | 34(63.0) | 180(73.8) | **0.02** |
| Yes | 326(39.7) | 66(23.2) | 64(26.2) | 196(66.7) | **<0.001** | 44 (23.2) | 20(37.0) | 64(26.2) | 0.40 |
| ^Wash wound with diluted povidone-iodine^  ksQ5 n (%) |  |  |  |  |  |  |  |  |  |
| No | 559(68.2) | 240(85.4) | 186(76.2) | 133(45.1) | **<0.001** | 148(77.9) | 38(70.4) | 186(76.2) | **0.058** |
| Yes | 261(31.8) | 41(14.6) | 58(23.8) | 162(54.9) | **<0.001** | 42(22.1) | 16(29.6) | 58(23.8) | 0.86 |

^a^Pian Upe Game Reserve; ^b^Queen Elizabeth NP, Kibaale NP & Katonga game reserve; ^c^Murchison Falls NP, ^k^Kamwenge

^“ksQ1: Does washing animal bite wound with water and soap help control rabies?”; “ksQ2: Does PEP with Human Rabies Immune Globulin (HRIG) at 0 day of exposure control rabies?”; “ksQ3: Does Pre-exposure vaccination for at risk people help control rabies?”; “ksQ4: Does Gentle wash/irrigation of wound in water help control rabies”, “ksQ5: Does Washing wound with diluted povidone-iodine help control rabies”^
