# Supplementary material for: Descriptive analyses of knowledge, attitudes, and practices regarding rabies transmission and prevention in rural communities near wildlife reserves in Uganda: a One Health cross-sectional study
Source: Trop Med Health. 2024 Jul 19;52:48. doi: 10.1186/s41182-024-00615-2 (PMC11264860; doi:10.1186/s41182-024-00615-2)
Supplement: Supplementary file 5 — Supplementary Material 5. [file 41182_2024_615_MOESM5_ESM.docx]

**Supplementary file, S5. Attitudes towards rabies transmission and prevention among households neighbouring national parks in Uganda**

|  |  | District | | |  | Education level^k^ | |  | p-value |
| --- | --- | --- | --- | --- | --- | --- | --- | --- | --- |
| Variable | N=843 | Bukedea (n=302) | Kamwenge (n=245) | Nwoya (n=296) | p-value | Primary & below (n= 619) | Post primary (n= 224) | N (%) |  |
| ^Think that a person bitten by a rabid dog should seek treatment from a health facility/veterinary facility^  bQ1 **n (%)** | N (%) |  |  |  |  |  |  |  |  |
| Agreed | 758(90.4) | 292(97.3) | 217(88.9) | 249(84.4) | 0.26 | 171(90.0) | 46(85.2) | 217(88.9) | **0.073** |
| Not sure | 79(9.4) | 8 (2.7) | 26(10.7) | 45(15.3) | **<0.001** | 18(9.5) | 8(14.8) | 26(10.7) | 0.63 |
| Disagree | 2(0.2) | 0(0.0) | 1(0.4) | 1(0.3) | 0.56^Ϯ^ | 1(0.5) | 0(0.0) | 1(0.4) | 0.55^Ϯ^ |
| ^Believe that communities are willing to vaccinate their pets/dogs^  bQ2 n (%) |  |  |  |  |  |  |  |  |  |
| Agreed | 779(93.0) | 287(95.4) | 223(91.4) | 269(91.8) | 0.84 | 173(91.1) | 50(92.6) | 223(91.4) | 0.16 |
| Not sure | 46(5.5) | 8(2.7) | 16(6.6) | 22(7.5) | **0.03** | 13(6.8) | 3(5.6) | 16(6.6) | 0.48^Ϯ^ |
| Disagree | 13(1.6) | 6(2.0) | 5(2.1) | 2(0.7) | 0.33 | 4 (2.1) | 1(1.9) | 5(2.1) | 0.74^Ϯ^ |
| ^do you think vaccination of dogs/pets greatly contributes to rabies control in your district^  bQ3 **n (%)** |  |  |  |  |  |  |  |  |  |
| Agreed | 803(95.6) | 298(99.0) | 232(95.1) | 273(92.5) | 0.72 | 180(94.7) | 52(96.3) | 232(95.1) | 0.15 |
| Not sure | 35(4.2) | 2(0.7) | 11(4.5) | 22(7.5) | **<0.001** | 9(4.7) | 2(3.7) | 11(4.5) | 0.53^Ϯ^ |
| Disagree | 2(0.2) | 1(0.3) | 1(0.4) | 0(0.0) | 0.57 | 1(0.5) | 0(0.0) | 1(0.4) | 0.55^Ϯ^ |
| ^Believe that community sensitization has not been sufficiently done in our community^  bQ4 **n (%)** |  |  |  |  |  |  |  |  |  |
| Agreed | 567(67.5) | 205(68.1) | 155(63.5) | 207(70.2) | 0.63 | 119(62.6) | 36(66.7) | 155(63.5) | 0.35 |
| Not sure | 61(7.3) | 7(2.3) | 18(7.4) | 36(12.2) | **<0.001** | 15(7.9) | 3(5.6) | 18(7.4) | 0.34^Ϯ^ |
| Disagree | 212(25.2) | 89 (29.6) | 71(29.1) | 52(17.6) | **0.005** | 56(29.5) | 15(27.8) | 71(29.1) | 0.30 |
| ^Believe that health centers should work closely with veterinary office to curb down rabies^  bQ5 **n (%)** |  |  |  |  |  |  |  |  |  |
| Agreed | 803(95.6) | 291(96.7) | 234(95.9) | 278(94.2) | 0.95 | 183(96.3) | 51(94.4) | 234(95.9) | 0.098 |
| Not sure | 27(3.2) | 4(1.3) | 9(3.7) | 14(4.8) | **0.06** | 6(3.2) | 3(5.6) | 9(3.7) | 0.65^Ϯ^ |
| Disagree | 10(1.2) | 6 (2.0) | 1(0.4) | 3(1.2) | 0.23^Ϯ^ | 1(0.5) | 0(0.0) | 1(0.4) | 0.55^Ϯ^ |

^k^Kamwenge; ^Ϯ^Fisher’s exact p-value

^bQ1: Think that a person bitten by a rabid dog should seek treatment from a health facility/veterinary facility; bQ2: Believe that communities are willing to vaccinate their pets/dogs; bQ3: do you think vaccination of dogs/pets greatly contributes to rabies control in your district; bQ4: Believe that community sensitization has not been sufficiently done in our community; bQ5: Believe that health centers should work closely with veterinary office to curb down rabies^
