# Supplementary material for: Descriptive analyses of knowledge, attitudes, and practices regarding rabies transmission and prevention in rural communities near wildlife reserves in Uganda: a One Health cross-sectional study
Source: Trop Med Health. 2024 Jul 19;52:48. doi: 10.1186/s41182-024-00615-2 (PMC11264860; doi:10.1186/s41182-024-00615-2)
Supplement: Supplementary file 6 — Supplementary Material 6. [file 41182_2024_615_MOESM6_ESM.docx]

**Supplementary file (S6). Practices towards rabies transmission and prevention among households neighbouring national parks in Uganda.**

|  |  | District | | |  | Education level^k^ | |  | p-value |
| --- | --- | --- | --- | --- | --- | --- | --- | --- | --- |
| Variable | N=843 | Bukedea (n=302) | Kamwenge (n=245) | Nwoya (n=296) | p-value | Primary & below (n= 619) | Post primary (n= 224) | N (%) |  |
| ^The grazing system used in this community/household^  **pQ3 n (%)** |  |  |  |  |  |  |  |  |  |
| paddock | 36(4.5) | 2(0.7) | 25(10.5) | 9(3.3) | **<0.001** | 20(10.9) | 5(9.3) | 25(10.5) | 0.46 |
| **tethering** | 159(20.0) | 80(28.5) | 42(17.7) | 37(13.4) | **<0.001** | 29 (15.8) | 13(24.1) | 42(17.7) | 0.52 |
| free range | 589(74.1) | 195(69.4) | 167(70.2) | 227(82.3) | 0.19 | 131(71.2) | 36(66.7) | 167(70.2) | 0.14 |
| other | 11(1.4) | 4(1.4) | 4(1.7) | 3(1.1) | 0.82 | 4(2.2) | 0(1.7) | 4(1.7) | 0.23^Ϯ^ |
| ^Do your animals graze on their own^  **pQ4 n (%)** |  |  |  |  |  |  |  |  |  |
| never | 570(67.6) | 214(70.9) | 201(82.0) | 155(52.4) | **<0.001** | 162(84.8) | 39(72.2) | 201(82.0) | **0.02** |
| less than 3times | 27(3.2) | 7(2.3) | 1(0.4) | 19(6.4) | **<0.001** | 1(0.5) | 0(0.0) | 1(0.4) | 0.55^Ϯ^ |
| 3 times | 43(5.1) | 12(4.0) | 18(7.4) | 13(4.4) | 0.18 | 11(5.80 | 7(13.0) | 18(7.4) | 0.24 |
| always | 203(24.1) | 69(22.9) | 25(10.2) | 109(36.8) | **<0.001** | 17(8.9) | 8(14.8) | 25(10.2) | 0.54 |
| ^How are your dogs kept?^  **pQ6 n (%)** |  |  |  |  |  |  |  |  |  |
| Caged | 12(1.4) | 0(0.0) | 6(2.5) | 6(2.0) | **0.032^Ϯ^** | 3(1.6) | 3(5.6) | 6(2.5) | 0.19^Ϯ^ |
| Chained | 56(6.6) | 0(0.0) | 15(6.1) | 41(13.9) | **<0.001^Ϯ^** | 12(6.3) | 3(5.6) | 15(6.1) | 0.56^Ϯ^ |
| Free-roaming | 775(91.9) | 302(100.0) | 224(91.4) | 249(84.1) | **0.13** | 176(92.2) | 48(88.9) | 224(91.4) | **0.08** |
| ^How often do your dogs hunt in the game reserve or national park^  **pQ7 n (%)** |  |  |  |  |  |  |  |  |  |
| never | 712(88.5) | 237(85.6) | 241(99.2) | 234(82.1) | **<0.001^Ϯ^** | 188(99.5 | 53(98.2) | 241(99.2) | 0.11 |
| less than 3times | 15(1.9) | 5(1.8) | 0(0.0) | 10(3.5) | 0.13^Ϯ^ |  |  |  |  |
| 3 times | 17(2.1) | 8(3.0) | 1(0.4) | 8(2.8) | 0.11^Ϯ^ | 0(0.0) | 1(1.9) | 1(0.4) | 0.10^Ϯ^ |
| always | 61(7.6) | 27(9.8) | 1(0.4) | 33(11.6) | **<0.001** | 1(0.5) | 0(0.0) | 1(0.41) | 0.55 |
| ^Are there any wild life attacks in your community^ per week  **pQ8 n (%)** |  |  |  |  |  |  |  |  |  |
| never | 568(68.4) | 189(64.7) | 168(68.9) | 211(71.8) | 0.42 | 131(69.0) | 37(68.5) | 168(68.9) | 0.18 |
| less than 3times | 43(5.2) | 22(7.5) | 12(4.9) | 9(3.1) | **0.07** | 10(5.3) | 2(3.7) | 12(4.9) | 0.44^Ϯ^ |
| 3 times | 46(5.5) | 4(1.4) | 30(12.3) | 12(4.1) | **<0.001** | 22(11.6) | 8(14.8) | 30(12.3) | 0.99 |
| always | 173(20.8) | 77(26.4) | 34(13.9) | 62(21.1) | **0.011** | 27(14.2) | 7(13.0) | 34(14.0) | 0.43 |

^k^Kamwenge; ^Ϯ^Fisher’s exact p-value

^pQ1: How often do you graze your animals in game reserves or national park; pQ2: How often do you graze your animals with dogs; pQ3: The grazing system used in this community/household^

^pQ4: Do your animals graze on their own; pQ5: How often do you vaccinate your dogs; pQ6: How are your dogs kept; pQ7: How often do your dogs hunt in the game reserve or national park.^

^pQ8: Are there any wildlife attacks in your community.^
